# Application of nanodisc technology for direct electrochemical investigation of plant cytochrome P450s and their NADPH P450 oxidoreductase

**DOI:** 10.1038/srep29459

**Published:** 2016-07-08

**Authors:** Krutika Bavishi, Tomas Laursen, Karen L. Martinez, Birger Lindberg Møller, Eduardo Antonio Della Pia

**Affiliations:** 1 Plant Biochemistry Laboratory, Department of Plant and Environmental Sciences, Thorvaldsensvej 40, DK-1871 Frederiksberg C, University of Copenhagen, Denmark; 2 VILLUM Research Center for Plant Plasticity, Thorvaldsensvej 40, DK-1871 Frederiksberg C, University of Copenhagen, Denmark; 3 Center for Synthetic Biology ‘bioSYNergy’, Thorvaldsensvej 40, DK-1871 Frederiksberg C, University of Copenhagen, Denmark; 4 Joint BioEnergy Institute, Feedstocks Division, Emeryville, CA 94608, USA; 5 Bio-Nanotechnology Laboratory, Department of Chemistry & Nano-Science Center, Universitetparken 5, DK-2100, University of Copenhagen, Denmark

## Abstract

Direct electrochemistry of cytochrome P450 containing systems has primarily focused on investigating enzymes from microbes and animals for bio-sensing applications. Plant P450s receive electrons from NADPH P450 oxidoreductase (POR) to orchestrate the bio-synthesis of a plethora of commercially valuable compounds. In this report, full length CYP79A1, CYP71E1 and POR of the dhurrin pathway in *Sorghum bicolor* were reconstituted individually in nanoscale lipid patches, “nanodiscs”, and directly immobilized on unmodified gold electrodes. Cyclic voltammograms of CYP79A1 and CYP71E1 revealed reversible redox peaks with average midpoint potentials of 80 ± 5 mV and 72 ± 5 mV vs. Ag/AgCl, respectively. POR yielded two pairs of redox peaks with midpoint potentials of 90 ± 5 mV and −300 ± 10 mV, respectively. The average heterogeneous electron transfer rate constant was calculated to be ~1.5 s^−1^. POR was electro-catalytically active while the P450s generated hydrogen peroxide (H_2_O_2_). These nanodisc-based investigations lay the prospects and guidelines for construction of a simplified platform to perform mediator-free, direct electrochemistry of non-engineered cytochromes P450 under native-like conditions. It is also a prelude for driving plant P450 systems electronically for simplified and cost-effective screening of potential substrates/inhibitors and fabrication of nano-bioreactors for synthesis of high value natural products.

Cytochromes P450 have been aptly nicknamed “nature’s blow torches” owing to their ability to catalyze an impressive diversity of complex and chemically unfavoured reactions with high specificity and stereo-selectivity. The catalytic cycle of P450s involves a complex redox cycle of the heme (Fe^3+^ ↔ Fe^2+^) with the formation of several intermediates^1^. The general reaction can be summed up as RH + O_2_ + 2H^+^ 2e^−^ → ROH + H_2_O. Though the cytochrome P450 reactions fundamentally follow the stoichiometry of 2 electrons: 1 dioxygen: 1 product, they are vulnerable to uncoupling branch points (Fig. 1), namely the (a) autoxidation (b) peroxide shunt and (c) oxidase shunt pathways which consume the reducing equivalents and hinder product formation^2^.

The eukaryotic microsomal P450s (Class II) are dependent on electrons provided by the membrane bound NADPH-dependent cytochrome P450 oxidoreductase (POR). POR belongs to the family of diflavin reductase enzymes, which harbor one molecule each of flavin adenine dinucleotide (FAD) and flavin mononucleotide (FMN). Upon binding of NAPDH, two electrons are transferred in the form of a hydride anion to the FAD and further to the FMN coenzyme. However, they are delivered to the P450s one by one at specific points of the catalytic cycle. Spectropotentiometric studies have deconvoluted four different redox couples of POR, *i.e*. FAD_ox_/FAD_sq_, FAD_sq_/FAD_red_, FMN_ox_/FMN_sq_ and FMN_sq_/FMN_red_^3^. In microsomes, the FAD_ox_/FMN_sq_ form occurs predominantly and is stabilized as the resting state. The redox cycling of flavins *in vivo* follows a 1-3-2-1 scheme^4^ (Supplementary Fig. S1) and overall directional flow of electrons is summarized as NADPH →FAD→FMN→P450. In animals and humans, a single gene encoding POR is present while plants typically harbor 3–4 POR homologs^5^, although the biological relevance of the presence of multiple POR isoforms has not yet been resolved^6^.

Plant P450s are highly diversified and play a pivotal role in the synthesis of a plethora of bio-active specialized metabolites which aid the sessile organisms to adapt to a variety of environmental challenges. Among the bio-active, specialized metabolites, many are commercially valuable compounds used as flavors, fragrances, colorants and pharmaceuticals^7^. To this date, several genetic and metabolic engineering approaches have been developed to bio-synthesize these compounds based on initial functional screening to identify the P450 enzymes involved. The repertoire of genomic sequences has been expanding substantially, currently counting more than 7000 sequences in the plant P450 database^8^. The immense number of P450 isoforms and their complex catalytic mechanisms renders it challenging to perform high throughput functional screening. Nevertheless, the foremost caveat in the exploitation of these P450-derived metabolites is that they are typically produced in extremely small quantities by plants. Direct extraction is a low yield-high cost enterprise while chemical synthesis of natural products with highly complex chemical structures is often not feasible. Furthermore, NADPH, the fundamental electron donating cofactor is expensive which lowers the potential for commercial exploitation. Recently, the dhurrin pathway was directly coupled to photosystem I (PSI) in the chloroplasts generating a light driven production system evading NADPH^9^,^10^,^11^. This approach utilized the ability of light-driven electron transport through PSI to reduce ferredoxin, which in turn served as electron donor to the P450 enzymes. Coupling photosynthetic and metabolic pathways holds extensive industrial potential for efficient and sustainable production systems for bio-active natural products from plants.

Another and yet rather unexplored possibility to circumvent the need to supplement plant P450 systems with NADPH is by electrochemistry. Thin film cyclic voltammetry has classically been the most favored electrochemical technique for evaluating redox states of the microbial and human P450 enzymes. The cyclic voltammograms examine voltage-current relationships of enzymes confined at an electrode surface offering discrete reversible oxidation and reduction peaks from which formal/midpoint potential values (E_m_) can be calculated. Over the years, this approach has gained momentum primarily in deciphering the characteristic redox features and electro-catalytic potential of cytochromes P450^12^. Specifically engineered enzymes genetically fused to redox proteins have been interfaced on a range of electrode surfaces employing various biomimetic systems and immobilization strategies in order to develop electrochemical biosensors^13^. Surprisingly, these studies have been restricted to crude microsomal fractions or detergent solubilized enzymes. However, detergents may have detrimental effect on the conformation of proteins and hinder enzymatic activity^14^,^15^. In contrast, nanodiscs could provide a native-like membrane environment for membrane proteins. They are highly monodisperse entities consisting of a patch of phospholipids encapsulated by a rim of membrane scaffold proteins (MSP) and have been the choice of biomimetic system for a diverse array of biophysical studies^16^,^17^,^18^.

Here we present a direct electrochemical study of purified full-length plant P450s and POR reconstituted in nanodiscs (Fig. 2), which should pave the way for exploring other plant based P450 systems. The dhurrin pathway of the crop plant *Sorghum bicolor* has been used as the model system^19^. It encompasses two P450s, CYP79A1 and CYP71E1, which accept electrons from POR and sequentially convert the aromatic amino acid tyrosine to (*E*)-*p*-hydroxyphenylacetaldoxime ((E)-oxime) and *p*-hydroxyphenylmandelonitrile (cyanohydrin), respectively^20^,^21^. Finally, the labile cyanohydrin is stabilized by glucosylation catalyzed by the soluble glycosyltransferase, UGT85B1, resulting in the formation of dhurrin (Supplementary Fig. S2 22). Upon attack by herbivorous chewing animals, dhurrin is brought in contact with β- glucosidases and hydrolyzed into the cyanohydrin, which either spontaneously or enzymatically decomposes into *p*-hydroxybenzaldehyde and HCN, the latter acting as defense compound against respiring animals^23^. In the current study, CYP79A1, CYP71E1 and POR were individually reconstituted in nanoscale lipid bilayer discs (nanodiscs) and solvent accessible cysteine residues were utilized to directly immobilize the proteins on unmodified gold electrodes. Cyclic voltammetry was employed to determine redox potentials and electron transfer rate constants. The electro-catalytic activity of enzymes was investigated.

## Experimental

### Materials

All chemicals were of analytical grade and purchased from Sigma-Aldrich (Denmark) unless otherwise stated. Ni-NTA Agarose and 2′5′ ADP Sepharose were purchased from Qiagen and GE Healthcare Life Sciences, respectively. Prepacked Size Exclusion Chromatography (SEC) column Superdex 200 HR 10/30 for high-resolution preparative separation was obtained from GE Healthcare Life Sciences. Phospholipids: 1, 2-dilauroyl-sn-glycero-3-phosphocholine (DLPC), 1, 2-dilauroyl-sn-glycero-3-phosphoglycerol (DLPG) and 1-palmitoyl-2-[12-((7-nitro-2-1, 3-benzoxadiazol-4-yl) amino) dodecanoyl]-sn-glycero-3-phosphocholine (NBD PC) were purchased from Avanti Polar Lipids (Alabaster, USA). Silica gel 60 TLC plates were bought from Merck Millipore (Denmark). Mini-Criterion TGX Stain-Free precast gels and Bio-Beads SM-2 were obtained from Bio-Rad. Radiolabeled [U-^14^C] tyrosine (50 μCi/mL, specific activity 450 mCi/mmol) was from Perkin Elmer whereas *p*-hydroxyphenylacetaldoxime (oxime) was obtained as previously reported^24^. Radiolabeled oxime was prepared enzymatically by reconstitution of purified CYP79A1 as described earlier^25^. All solutions were prepared in Milli-Q system purified water.

### Protein Expression and Purification

Large-scale expression and purification of the full-length membrane proteins, CYP79A1, CYP71E1 and POR (isoform POR2b) from *Sorghum bicolor* was conducted as described in detail elsewhere^26^. Purified proteins were flash-frozen and stored at −80 °C in buffer [50 mM Tris (pH 7.4), 100 mM NaCl, 0.1% TritonX-100 and 20% glycerol]. The membrane scaffold protein, MSP1E3D1 was expressed and purified according to the standard protocol^27^. Purity of all proteins was assessed by PAGE using pre-cast TGX stain free gels (12%) and scanned by the Gel Doc EZ System (Bio-Rad).

### Nanodisc reconstitution of proteins

Purified proteins were reconstituted individually into nanodiscs (NDs) containing the following lipid mixture DLPC: DLPG: NBD-PE (73/25/2 mol %) and the membrane scaffolding protein MSP1E3D1. Detergent removal was facilitated by incubation with Bio-Beads for 4 h. Molar ratios of MSP: Lipid: Protein was used as follows: 1:120:0.1, resulting in approximately five-times molar excess of nanodiscs to P450 or POR enzyme. During the reconstitution of POR, 1 μM FMN and 1 μM FAD were included to replenish the loss of flavin coenzymes. Empty nanodiscs (containing no membrane proteins) were prepared for control experiments. Purification of nanodiscs was achieved by size exclusion chromatography (SEC) on a preparative HPLC (Shimadzu) equipped with a Superdex 200 HR 10/30 column (flow rate: 0.5 mL/min). Protein-loaded nanodisc fractions were selected, combined and stored at −80 °C until use. No effects of flash freezing on structural and functional integrity of nanodiscs were observed^28^.

### Enzyme assays

The catalytic activity for detergent solubilized and nanodisc reconstituted POR was measured by the standard cytochrome-*c* assay. Briefly, the enzyme in buffer containing Tris 50 mM (pH 7.4) and 100 mM NaCl was incubated with 50 μM cytochrome *c* in a 1 mL cuvette for 2–3 min. After recording a baseline, the reaction was initiated by addition of 1 mM NADPH and the amount of cytochrome c reduced was monitored by the time-dependent increase in absorbance at 550 nm by a Perkin-Elmer Lambda 650 UV/V is spectrophotometer (extinction coefficient = 21.2 mM^−1^ cm^−1^). The slope of the curve in the linear region was used to determine the catalytic activity as described earlier^29^.

Radioactivity assays were used to measure activity of the cytochromes P450^26^. The 30 μL assay mixture contained 5 μL CYP79A1 or CYP71E1 (concentration ∼1–3 μM), tyrosine or *p*-hydroxyphenylacetaldoxime (oxime) (100 μM), 2 μL L-[U-^14^C] tyrosine/oxime and 5 mM NADPH. Detergent solubilized POR (5 μM) was used as the electron donor for the purified P450s. For CYP79A1 and CYP71E1 in nanodiscs, soluble electron donors ferredoxin (8.3 μM) and ferredoxin NADP^+^ reductase (FNR) (0.6 μM) were used. Following incubation (30 min, 30 °C, 350 rpm), the formation of radioactively labeled oxime or *p*-hydroxybenzaldehyde (aldehyde) was monitored by application of a 10 μL aliquot on TLC plates and separated using a solvent system containing toluene, ethyl acetate, and methanol (30:8:1 v/v/v) as a mobile phase. TLC plates were dried and exposed to phosphor-imaging screens. A Storm 860 molecular imager equipped with ImageQuant 5.0 software was used to visualize the products (Amersham Biotech–Molecular Dynamics).

### Membrane protein film preparation

A 1.6 mm diameter disk gold electrode (MF-2014, BASi) was rinsed in deionized water and ethanol before being etched in hot piranha solution (3:1 mixture of H_2_SO_4_ and H_2_O_2_-Caution!) for 20 min. The electrode was thoroughly rinsed in deionized water and buffer A (50 mM potassium phosphate buffer pH 7.4 and 100 mM NaCl). The clean electrode was immersed into a 100 μL solution containing nanodiscs (1 μM) and incubated for 90 min at room temperature. Prior to measurements, the protein-functionalized electrode was rinsed with deionized water and buffer A in order to remove unbound proteins.

### Fluorescence microscopy and electrochemistry experiments

A Leica DM5500 B upright optical microscope with epifluorescence optics was used to image gold electrodes (IDA-1 chip from Micrux) functionalized with the nanodisc-reconstituted proteins. The microscopy images were acquired by excitation at 470/40 nm and emission at 525/50 nm. Data were analyzed using ImageJ software.

Electrochemical cyclic voltammetry experiments were performed on a CH Instrument (CHI630B) electrochemical analyzer connected to a three-electrode cell. The electrochemical cell consisted of a conventional single compartment containing 2 ml electrolyte (buffer A) and a three-electrode system. The functionalized gold electrode was used as working electrode, a platinum mesh as counter electrode and an Ag/AgCl in 3 M KCl as reference electrode (Eo = + 196 mV *vs* NHE). All potentials reported in this work are with reference to the Ag/AgCl electrode. The solution was saturated with a flow of nitrogen gas for at least 30 min before starting the experiments and nitrogen was continuously passed over the solution unless mentioned otherwise.

### Electro-driven catalysis

Electro-driven catalytic measurements of nanodiscs were performed in fully oxygenated conditions. The assay involved addition of 100 μM unlabeled tyrosine/oxime and 50 μL L-[U-^14^C] tyrosine/oxime as substrates for CYP79A1 and CYP71E1, respectively. The reaction was allowed to proceed for 20 min with slow magnetic stirring, after which the entire electrolytic solution (2 mL) was vacuum-concentrated (Thermo Scientific SpeedVac) to 20 μL, and applied to a TLC plate. Analysis of products was performed as described above. The formation of H_2_O_2_ was measured by the FOX 1 method^30^.

POR activity was studied by reduction of the pre-fluorescent substrate, resazurin, which is converted to the highly fluorescent product, resorufin^18^. Production of resorufin was measured by fluorescence (Excitation: 572 nm, Emission: 585 nm). Empty nanodiscs were used as controls for all activity assays.

## Results and Discussion

### Protein purification and preparation of nanodiscs

Codon optimized genes encoding full length CYP79A1 and CYP71E1 with a 6x his-tag at the C-termini and cloned into the pYeDP60 vector were expressed in *S. cerevisiae* BY4741 strains and purified by Ni-NTA affinity chromatography. The POR gene cloned in the pET-52b vector was heterologously expressed using *E. coli* BL21DE3 as host and affinity purified using 2′,5′-ADP Sepharose. Purified CYP79A1, CYP71E1 and POR were reconstituted in nanodiscs containing a mixture of phospholipids (DLPC, DLPG, and NBD-PC) and assembled using the membrane scaffold proteins MSP1E3D1. Addition of 20–25% anionic phospholipids (DLPG) is known to promote electron flow^31^ and facilitate insertion into membranes^32^. The fluorescent lipid, NBD PC was included to allow visualization of nanodiscs binding to the electrode surface. The scaffold protein MSP1E3D1, yielding nanodiscs with a 12.9 nm diameter, was preferred over MSP1D1, yielding 9.7 nm nanodiscs, owing to their enhanced stability^18^. For the first time, CYP79A1 and CYP71E1 were embedded into nanodiscs whereas preparation of POR nanodiscs has been published earlier^18^,^28^,^33^.

**Figure 3:**
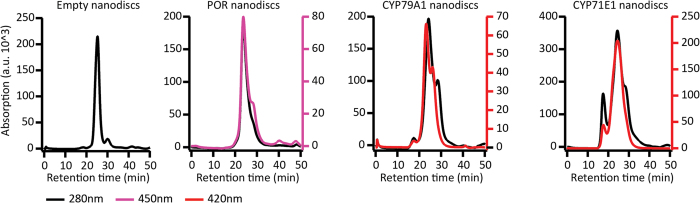
Chromatograms of nanodiscs after purification by size exclusion chromatography.

**Figure 4:**
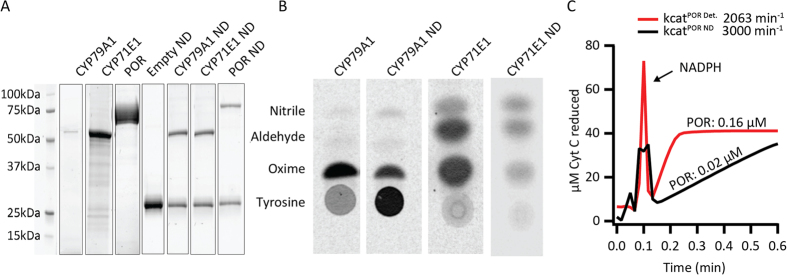
(**A**) TGX (Tris-Glycine eXtended) stain free gel showing purified (detergent solubilized) proteins and nanodisc (ND) reconstituted proteins (visualized by ImageLab software). (**B**) Radiolabeled activity assays of purified and ND reconstituted CYP79A1 and (Lane 1 and 2) where tyrosine is added as substrate and the enzymatically produced oxime is detected. For purified and reconstituted CYP71E1 (Lane 3 and 4), the substrate oxime is converted to aldehyde. *p*- Hydroxyphenylacetonitrile, a known biosynthetic intermediate produced by CYP71E1 is observed above the position of the oxime. (**C**) Cytochrome *c* assays for purified (detergent solubilized POR) and POR ND. The turnover rate was measured by determining the slope in the linear region of the curve. (The peak at 0.1 min is due to addition of NADPH for reaction initiation).

Upon reconstitution into nanodiscs and HPLC purification, relevant fractions were identified by monitoring their absorbance at 280 nm (empty nanodiscs), 280 nm/420 nm (P450s) and 280 nm/450 nm (POR) (Fig. 3) and analyzed by PAGE (Fig. 4A).

Detergent solubilized and nanodisc reconstituted P450s were shown to be functional in activity assays where the CYP79A1 catalyzes conversion of its substrate L-tyrosine to oxime and CYP71E1 catalyzes conversion of oxime to the product *p*-hydroxymandelonitrile (cyanohydrin). *In vitro*, the cyanohydrin spontaneously dissociates to *p*-hydroxybenzaldehyde (aldehyde). Incomplete turnover of *p*-hydroxyphenylacetaldoxime results in release of the *p*-hydroxyphenylacetonitrile (nitrile) (Fig. 4B). The activity of nanodiscs containing CYP79A1/CYP71E1 may seem reduced when compared to the detergent solubilized version. This is due to utilization of non-native soluble electron donors Fd/FNR. FNR is prone to inhibition by NADP^+^ formed by oxidation of NADPH. Full length, purified POR was not employed as the redox partner in these assays as inclusion of detergent micelles are known to destabilize nanodiscs. POR activity was monitored by reduction of cytochrome *c*. The detergent solubilized and nanodisc reconstituted POR displayed turnover rates of 2063 and 3000 min^−1^, respectively, which are comparable to previously published values^26^ (Fig. 4C).

### Surface immobilization of nanodiscs

The 3D homology model structures of CYP79A1, CYP71E1 and POR^34^ were assessed by the ASA view online server to determine the presence of solvent accessible cysteine residues^35^ (Supplementary Fig. S3). These were exploited to specifically bind the enzymes directly to the bare gold electrodes *via* thiol-gold chemistry^36^. Immobilization of the enzymes onto the gold surface was confirmed by epifluorescence imaging of thin gold electrodes functionalized with enzymes reconstituted nanodiscs containing fluorescently-labelled lipids (Supplementary Fig. S4).

**Figure 5:**
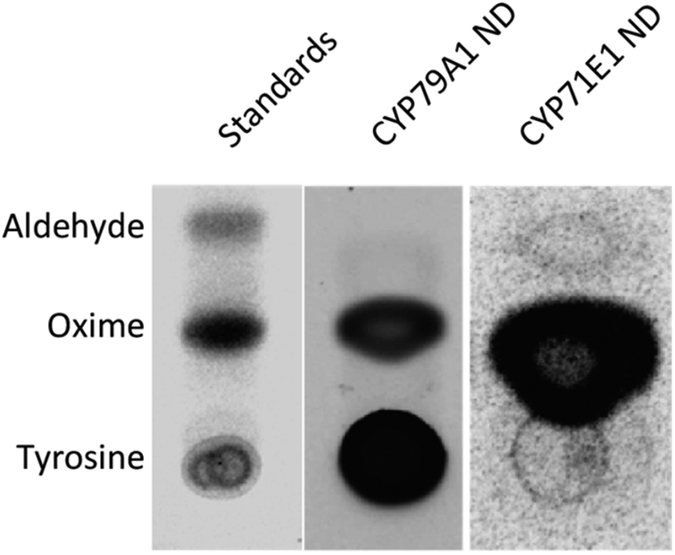
Bioconversion of substrates to products by electrode immobilized nanodiscs (ND) in presence of NADPH as the biological electron donor and Fd and FNR as carrier proteins. Oxime is formed from CYP79A1 and aldehyde is generated from the CYP71E1 containing nanodiscs.

The functional effect of direct immobilization of enzymes via multiple cysteine residues on unmodified electrodes was also investigated. The bound CYP79A1 and CYP71E1 nanodiscs were incubated in a vial containing 100 μL assay mixture consisting of the substrate (tyrosine or oxime) and biological electron donors and carriers (Ferredoxin, Ferredoxin NADP^+^ reductase and NADPH). TLC results showed formation of products (Fig. 5). These experiments served to ascertain that the enzymes maintained their catalytic properties while anchored on the gold electrode.

### Direct electrochemistry

**Figure 6:**
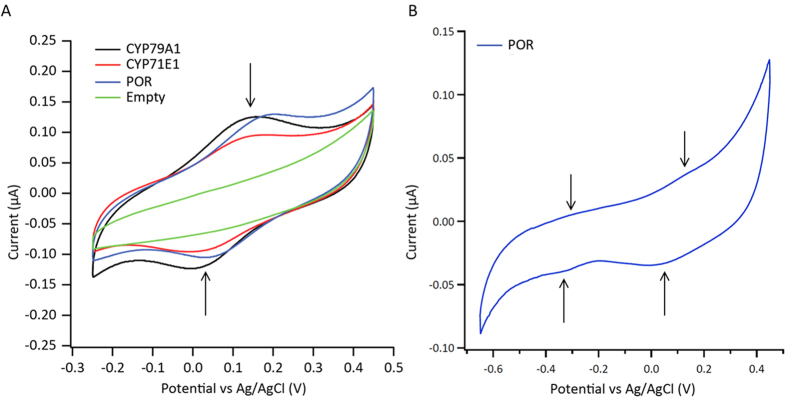
(**A**) Cyclic voltammograms recorded for the different proteins. Scan rate: 10 mV/s. (**B**) Cyclic voltammogram of POR reveals two pairs of peaks.

**Figure 7:**
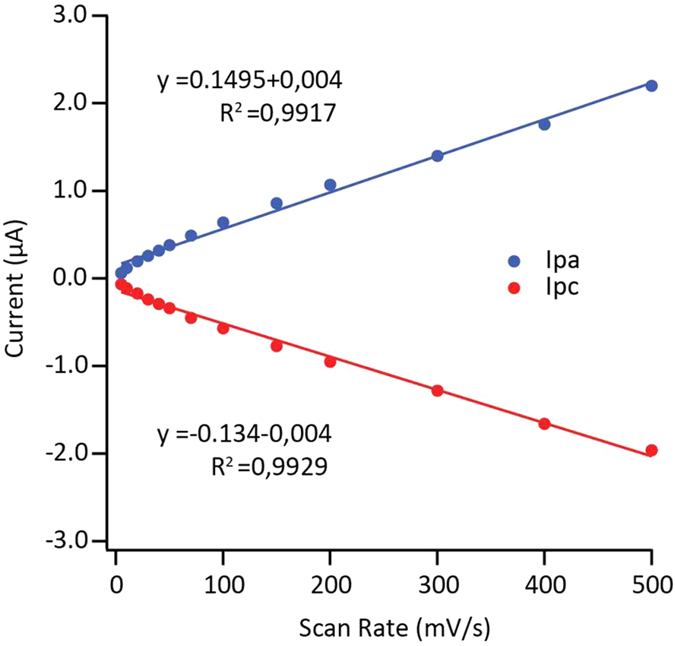
Relationship between peak anodic (Ipa) and peak cathodic currents (Ipc) as a function of scan rate (mV/s). The dots are raw data and solid lines represent the lines of best fit.

Cyclic voltammetry (CV) of the gold electrodes functionalized with the different proteins was recorded in a nitrogen-saturated atmosphere at a scan rate of 10 mV/s (Fig. 6A). An electrode incubated with empty nanodiscs (no protein) was included as negative control. The CV measurements displayed well-defined reversible pair of redox peaks corresponding to Fe^3+/^Fe^2+^ interconversion centered at 80 ± 5 mV and 72 ± 5 *vs*. Ag/AgCl for CYP79A1 and CYP71E1, respectively. POR revealed a stable and reversible redox peak at 90 ± 5 mV and a peak at around −300 mV (Fig. 6B) which corresponds to the FMN_ox/sq_ and a combination of the FAD_ox/sq_, FAD_sq/red_ and FMN_sq/red_ redox couples, respectively. The latter set of signals was not stable and lost within 2–3 cycles. No voltammetric response was observed in the absence of enzymes or with empty nanodiscs which indicated the absence of any other redox-active species in the system. All proteins remained stable and allowed the recording of multiple redox cycles. The anodic and cathodic current maxima (Ipa and Ipc) were linearly dependent on the scan rate (ν) up to 500 mV/s (Fig. 7) indicating that the response was due to a thin layer of surface-absorbed molecules and not diffusion limited.

The average apparent surface coverage was calculated by using the formula:


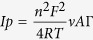


Where I*p* is the peak current the voltammetric peaks shown in Fig. 6A, n is the number of electrons transferred (1 for our experiments), F is the Faraday constant, R is the gas constant, T is the absolute temperature (K), ν is the scan rate (V s^−1^), A is the area of the working electrode (1.6 mm electrode diameter = 0.02 cm^2^ electrode area) and Γ is the surface coverage of the redox species (mol cm^−2^). The calculated value was found to be (1.4 ± 0.2) *10^14^ molecules/cm^2^. Considering a diameter of the scaffold protein in nanodiscs of ~13 nm, multiple layers of enzymes should be electroactive (~180). This theoretical value might indicate that (i) the enzymes are densely packed on the electrode surface and (ii) the enzymes (besides being specifically bound) could also be physisorbed in multiple layers. Interestingly, it was found that the anodic and cathodic peaks had approximatively the same area demonstrating reversible transfer of a single electron to the heme redox center of the P450s. The electron transfer rate constants were determined by fitting the Laviron’s equations for diffusion-less thin-layer voltammetry^37^ (Table 1).

The redox potentials of the reconstituted proteins in this study were positively shifted as compared to previously published values^38^,^39^,^40^. Positive shifts have been previously reported for cyclic voltammetry of P450cam in bio membrane-like films and attributed to lipid-protein interactions or electric double layer effects of lipids^41^. Das *et al*. have also reported an anodic shift of ~100 mV in spectropotentiomentry (in solution) measurements of nanodiscs containing rat POR^15^ and CYP3A4^42^. It is well established that surface confinement of cytochromes P450 tends to result in positively shifted values as compared to potentiometric titrations. Hence, this study highlights the role of the hydrophobic membrane environment and surface immobilization in regulating the redox potential of enzymes. The majority of previous electrochemical investigations have utilized surfactant films for immobilization of proteins on electrode surfaces. Interestingly, the redox potential obtained from direct electrochemistry of immobilized non-symbiotic plant hemoglobin class II from *Beta vulgaris*^43^ in the absence of any electron mediators was reported to be 171 mV (*vs* NHE). This is a significant anodic shift as compared to other hemoglobins (−100 to −126 mV)^44^,^45^ indicating the midpoint potentials of heme proteins can vary considerably. The underlying phenomenon for this shift was not discussed in detail. Nevertheless, these investigations provide credibility to the data in this study where enzymes of plant origin (P450s and POR) were subjected to direct electron transfer without mediators.

### Electro-driven catalysis

**Figure 8:**
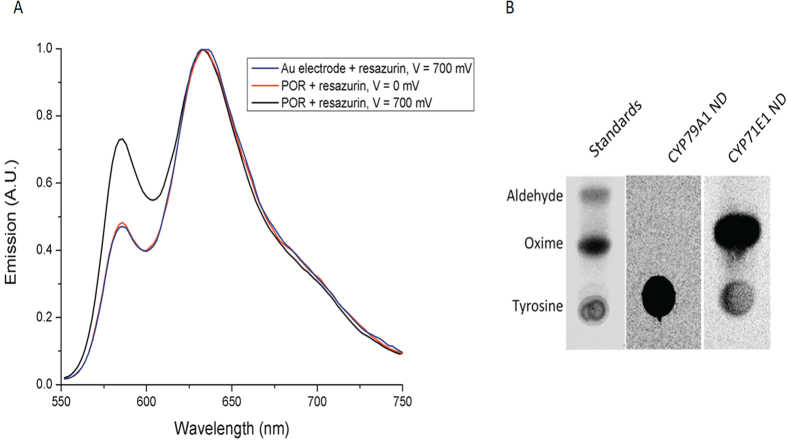
Electro-catalytic activity of enzymes (**A**) POR ND immobilized on the gold electrode. Excitation of the electrochemical solution was performed at 535 nm and emission recorded at 585 nm. (**B**) CYP79A1 and CYP71E1 ND tested in the presence of their substrates tyrosine and oxime, respectively. No product formation was detected.

As electron transfer from the gold electrode to the enzymes was observed, electrochemically-driven catalytic reactions were also investigated. POR activity was monitored by reduction of resazurin to the highly fluorescent product resorufin^18^. The fluorescence emission spectrum of the electrochemical solution was measured in presence of the substrate resazurin (10 μM) (Fig. 8A). A voltage-dependent signal at 590 nm was observed, demonstrating that electrons could be directly delivered from the electrode to POR and drive the catalytic cycle. However, due to low turnover values the apparent K_m_ for the enzymatic reaction could not be determined.

In order to examine the ability of immobilized CYP79A1 and CYP71E1 to carry out electro-catalytic based substrate conversions, a constant potential of −0.3 V *vs* Ag/AgCl was applied to the electrodes in air-saturated conditions in presence of tyrosine and oxime, respectively. Cyclic voltammetry revealed no shift in mid-point potential upon substrate addition. Although the FOX 1 assay confirmed the formation of hydrogen peroxide (H_2_O_2_) (Supplementary Fig. S5), physiological products were not detected (Fig. 8B). This indicated that the channeling of electrons directly from the gold electrode steers the P450 catalytic cycle, but is not efficient in driving it towards product formation. A possible explanation for this could be the dominance of uncoupling reactions/shunt pathways (e.g. formation of hydrogen peroxide)^1^,^46^. The peroxide uncoupling pathway extracts electrons to yield reduced oxygen products without substrate metabolism. Also, utilization of unmodified electrodes for immobilization could lead to poor relay of electrons. It has been proposed that gradual delivery of electrons can facilitate higher coupling rates and more efficient enzymatic turnover^47^. Several studies have shown the importance of the “molecular lego” approach wherein genetically fused constructs of flavodoxin (FMN containing electron transfer protein) and P450s are utilized^13^,^48^.

Alternatively, co-reconstitution of P450s and POR in nanodiscs could provide a more efficient electron transfer system given the activity observed for POR (Fig. 8A). However, ensuring a homogenous preparation with 1:1 stoichiometry is challenging due to size constraints of the nanodisc and difficulties in purification. Additionally, in such nanodiscs, the P450 and POR could be anchored to either of the phospholipid leaflet of the nanodisc, which may prevent their communication thereby inhibiting catalysis.

Recently, microsomes containing both the POR and cytochromes P450 have been employed^49^. These strategies attempted to mimic the natural electron flux *via* a cognate redox protein and have been largely successful.

## Conclusions

Since the first direct electrochemistry report of P450cam by Hill *et al*. in 1996^50^, studies of P450 enzymes have been restricted to enzymes of microbial and human origin with focus on their fundamental application in xenobiotic degradation and drug metabolism^38^. Here, a P450 system of plant origin was electrochemically studied following reconstitution of CYP79A1 and CYP71E1 into nanodiscs. The experimental set up utilized here was simple and cost-effective. The enzymes or the electrodes were not subjected to tedious engineering/modifications or embedment into surfactants that might be deleterious to proteins. The solvent accessible cysteine residues in the proteins were directly used for binding to the gold surfaces. This allowed direct electron transfer without electrode modification or the involvement of electron relaying protein moieties. CYP79A1, CYP71E1 and POR from the dhurrin pathway of *Sorghum bicolor* were functionally reconstituted into nanodiscs to probe electrochemical behavior in native-like environment of the enzymes. All proteins retained enzymatic activity upon binding to bare gold electrodes. The cyclic voltammetry measurements could be performed repeatedly and reproducibly. The reversible cyclic voltammograms of the enzymes displayed clearly resolvable redox peaks without the need for background subtraction, which is very often a challenge. The positive shift in redox potential is envisioned to be a combined manifestation of the presence of the hydrophobic environment provided by the nanodiscs, the immobilization technique used and absence of electron mediators. Deconvolution of the contribution of these factors was beyond the scope of this study. The electro catalytic conversion of substrates to products was accomplished for POR while CYP79A1 and CYP71E1 generated hydrogen peroxide but no products, which re-iterates the need for incorporation of spacer molecules and electron transfer enzymes (ferredoxin/POR) for driving the complex P450 catalytic mechanisms effectively. However, the focus of this report was to demonstrate the viability and incentives for conducting electrochemical examination employing this novel set-up and encouraging further studies on plant P450 electrochemistry.

This platform could be further optimized for product-driven electron flux and developed as an invaluable tool for identification of candidate P450 enzymes involved in biosynthesis of high value natural products^51^. Furthermore, an electronic device tethering the versatile P450s of metabolic pathways conjoined to a LC-MS apparatus could be constructed. This would orchestrate combinatorial biosynthesis and identification of high value plant products^52^. It would be intriguing to probe and compare the redox properties of the various homologs of POR to gain insights into their mechanistic role in plant adaptation. Recently, the diverse fields of cytochrome P450 electrochemistry and microfluidics have been integrated which opens up an array of biotechnological applications^53^. Such a multiplexed instrumentation could facilitate screening of substrates/inhibitors and small-scale synthesis of natural products^54^,^55^. The bottlenecks of purification and reconstitution of P450s can be averted by expressing the P450 enzymes on the cell surface^56^. Such whole cell direct biocatalysts could be directly immobilized as a “lab-on-chip” prototype^57^.

## Additional Information

**How to cite this article**: Bavishi, K. *et al*. Application of nanodisc technology for direct electrochemical investigation of plant cytochrome P450s and their NADPH P450 oxidoreductase. *Sci. Rep*. **6**, 29459; doi: 10.1038/srep29459 (2016).

## Supplementary Material

Supplementary Information

## Figures and Tables

**Figure 1:**
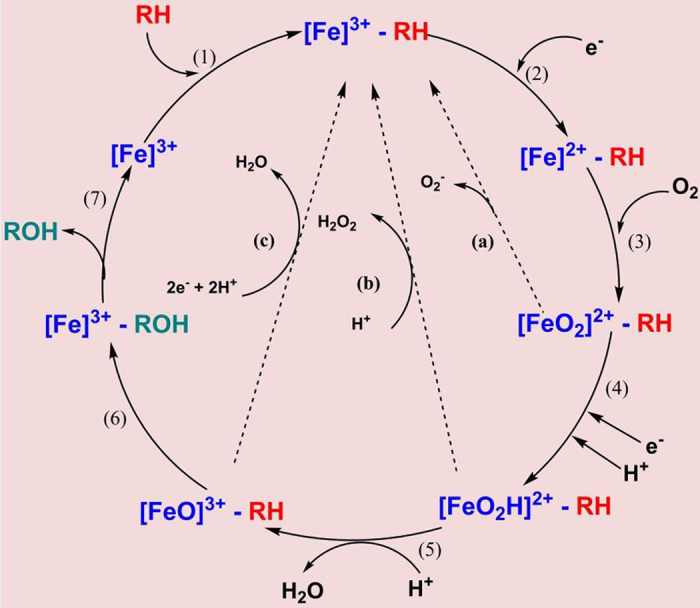
The catalytic cycle of cytochrome P450 with its branchpoints/shunt pathways. Various intermediate states are formed during the oxidation of substrate (RH) to its final product (ROH).

**Figure 2:**
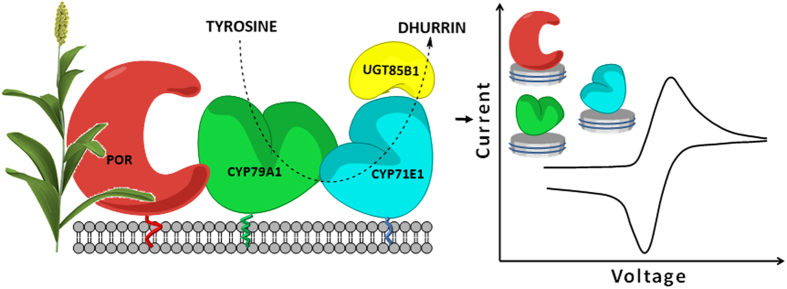
A diagrammatic representation of the Dhurrin pathway in the sorghum plant and bioelectrochemical analysis of its enzymes reconstituted in nanodiscs. (3D rendered sorghum plant © 3drenderings | Dreamstime.com).

**Table 1:** Redox and electron transfer parameters of the enzymes immobilized on the gold electrode surfaces.

Enzyme	E_red_ (mV)	E_ox_ (mV)	G (molecules/cm^2^)^i^	Electron transfer rate constant (s^−1^)^ii^
CYP79A1	−10	150	160*10^12^	1.4
CYP71E1	5	150	140*10^12^	1.8
POR	20	200	130*10^12^	1.6

^*i*^Error is ~10%.

^*ii*^The electron transfer rate constant was evaluated from curves collected at scan rate of 500 mV/s. Error is ~15%. The errors are the standard deviation of the mean evaluated on at least three independent measurements.
