# Supplementary material for: Application of nanodisc technology for direct electrochemical investigation of plant cytochrome P450s and their NADPH P450 oxidoreductase
Source: Sci Rep. 2016 Jul 8;6:29459. doi: 10.1038/srep29459 (PMC4937447; doi:10.1038/srep29459)
Supplement: Supplementary Information [file srep29459-s1.pdf]

Supplementary Information

Application of nanodisc technology for direct electrochemical investigation of plant cytochrome P450s and their NADPH P450 oxidoreductase

Krutika Bavishi[1, 2, 3], Tomas Laursen[1, 2, 3, 4], Karen L. Martinez[3, 5], Birger Lindberg Møller [1, 2, 3, *], Eduardo Antonio Della Pia[5]

[1]Plant Biochemistry Laboratory, Department of Plant and Environmental Sciences, Thorvaldsensvej 40, DK-1871 Frederiksberg C, University of Copenhagen, Denmark

[2]VILLUM Research Center for Plant Plasticity, Thorvaldsensvej 40, DK-1871 Frederiksberg C, University of Copenhagen, Denmark

[3]Center for Synthetic Biology 'bioSYNergy', Thorvaldsensvej 40, DK-1871 Frederiksberg C, University of Copenhagen, Denmark

[4]Joint BioEnergy Institute, Feedstocks Division, Emeryville, CA 94608, USA

[5] Bio-Nanotechnology Laboratory, Department of Chemistry & Nano-Science Center, Universitetparken 5, DK-2100, University of Copenhagen, Denmark

*Correspondence and request for materials should be addressed to blm@plen.ku.dk

Figure S1. Redox cycling in the FAD and FMN cofactors of POR via the "1-3-2-1" scheme.

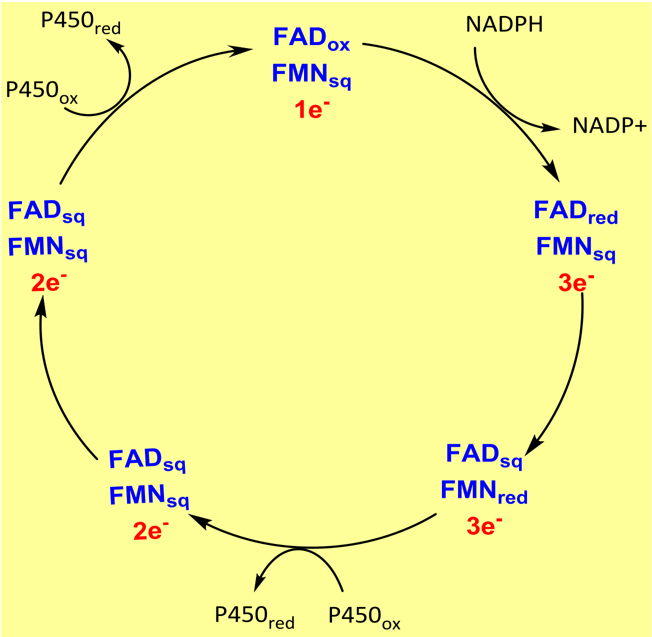

Figure S2. The Dhurrin biosynthetic pathway in *Sorghum bicolor* catalyzed by the cytochromes P450, CYP79A1 and CYP71E1 in the presence of the NADPH dependent cytochrome P450 oxidoreductase (POR). Adapted with permission from [1]

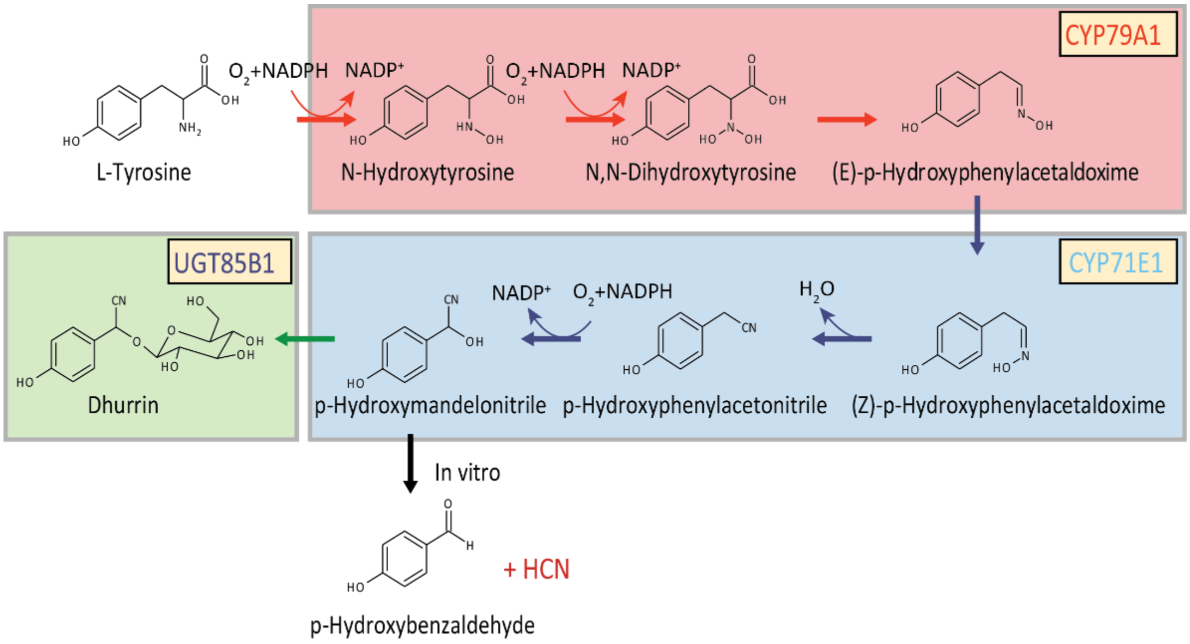

Calculation of electron transfer rate constant

The k_{ET} can be determined by using the Laviron's equation:

$$k_{ET} = \frac{\alpha n F \nu_c}{RT}$$

Where k_{ET} is rate of electron transfer at zero driving force, F is the Faraday constant, R is the gas constant, T is the absolute temperature (K), n is the number of electrons transferred (1 for our experiments), ν is the scan rate and the transfer coefficient, α, is determined by plotting the peak potential Ep against the log ν

Experimental

Immobilization of protein on electrode

In order to immobilize nanodiscs on the gold electrode, the sequences of CYP79A1, CYP71E1 and POR were analyzed for presence of cysteine residues. The relative solvent accessibilities of the residues were assessed by rendering the solvent accessible surface of the 3D homology models in PyMol. The online server, http://www.abren.net/asaview/ was also utilized[2]. Based on these findings, the cysteines with surface exposed thiols were determined (**Fig. S4**). The immobilization via thiol-gold chemistry was confirmed by epifluorescence imaging (**Fig. S5**)

Figure S3. A diagrammatic representation of surface accessible cysteine residues of the CYP79A1 (A, B), CYP71E1 (C, D) and POR (E). The cysteine residues are colored in orange.

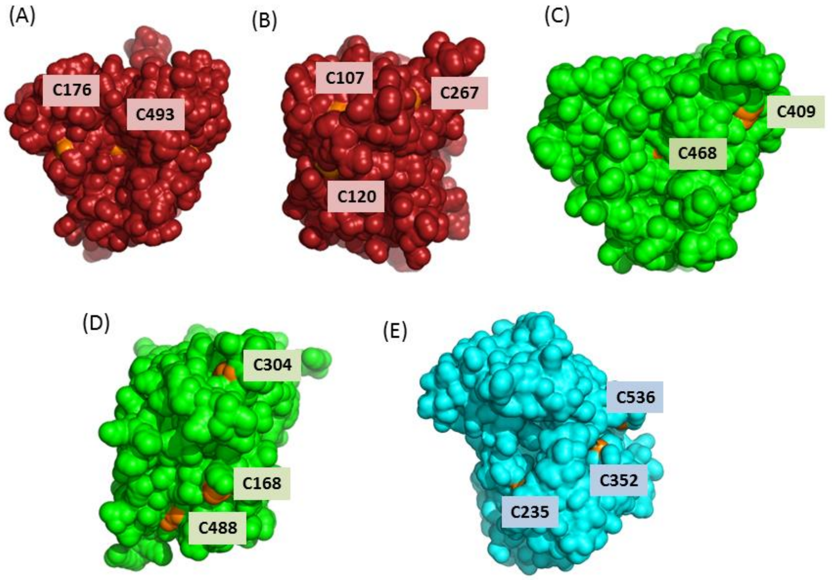

Figure S4. DIC (left) and GFP (right) channel images of gold electrodes from Micrux functionalized with CYP79A1 in nanodiscs containing NBD-tagged lipids. In the DIC image the gray stripes are 10 µm-wide gold electrodes and the black stripes are Pyrex. Nanodiscs containing NBD-lipids localized selectively onto the gold electrodes (right). These are false-color images acquired with the excitation and emission filter settings being Ex470/40 and Em525/50. Scale bar is 20 µm.

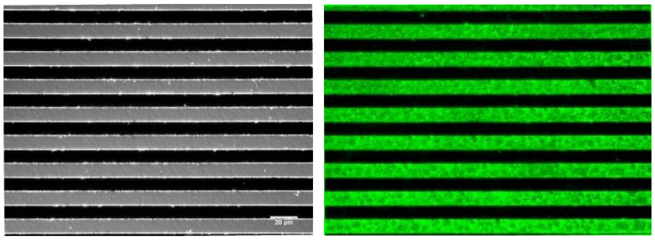

FOX 1 assay for detection of hydrogen peroxide

The FOX 1 assay is based on the oxidation of Fe^{2+} by peroxide under acidic condition, followed by the reaction of Fe^{3+} with the dye, xylenol orange[3]. The working reagent is prepared freshly before use and consists of 500 mM ammonium ferrous sulfate, 50 mM H_2SO_4, 200 mM xylenol orange and 200 mM sorbitol. The FOX 1 assay was used to qualitatively assess the release of hydrogen peroxide during electro-catalysis of CYP79A1. After addition of the substrate (100 µM tyrosine) to the immobilized CYP79A1 and application of potential (-300mV) for 20 minutes, 500 µL of the electrochemical solution was mixed with 500 µL of the freshly prepared assay reagent. The mixture was incubated for 45 minutes to determine the release of H_2O_2. Control assays were performed similarly.

Figure S5. A FOX 1 reagent (vial 4) mediated determination of H_2O_2 release during electro-catalysis by CYP79A1 nanodiscs. A visible color change of the solution from yellow to purple occurs (vial 3). Negligible color development occurs in the absence of enzyme or substrate (vial 1 and 2).

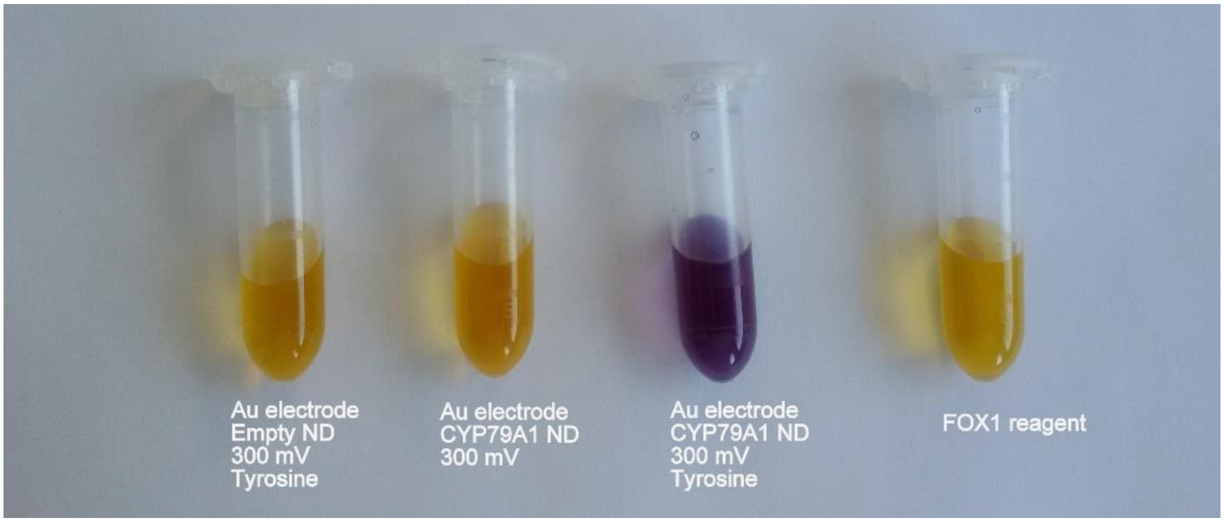

References

1. Laursen, T.; Moller, B. L.; Bassard, J. E., Plasticity of specialized metabolism as mediated by dynamic metabolons. *Trends Plant Sci.* **2015,** *20* (1), 20-32.

2. Ahmad, S.; Gromiha, M.; Fawareh, H.; Sarai, A., ASAView: Database and tool for solvent accessibility representation in proteins. *BMC Bioinformatics* **2004,** *5* (1), 1-5.

3. Jiang, Z.-Y.; Woollard, A. C. S.; Wolff, S. P., Hydrogen peroxide production during experimental protein glycation. *FEBS Lett.* **1990,** *268* (1), 69-71.
